# Role of Therapeutic Plasma Exchange in Severe Hypertriglyceridemia-Induced Acute Pancreatitis Associated With Preeclampsia: A Case Report

**DOI:** 10.7759/cureus.113485

**Published:** 2026-07-27

**Authors:** Hossam Algallie, Fatima Elamin, Sana Saleem, Abdelrahman Balal, Nissar Shaikh

**Affiliations:** 1 Anesthesiology and Critical Care, Hamad Medical Corporation, Doha, QAT; 2 Medical Education, Hamad Medical Corporation, Doha, QAT

**Keywords:** acute pancreatitis, hypertriglyceridemia, plasmapheresis, pregnancy, severe preeclampsia

## Abstract

Acute pancreatitis during pregnancy is an uncommon but serious condition associated with significant maternal and fetal morbidity. Hypertriglyceridemia-induced acute pancreatitis (HTG-AP) is a severe etiology that presents unique diagnostic and management challenges, particularly when complicated by preeclampsia. Diagnosis may be difficult because clinical manifestations can overlap with those of other obstetric emergencies.

We report the case of a 30-year-old woman at 33 weeks’ gestation with a history of type 2 diabetes mellitus and chronic hypertension who presented with severe abdominal pain, headache, hypertension, and proteinuria. Laboratory investigations demonstrated severe hypertriglyceridemia (>50 mmol/L) and elevated pancreatic enzymes, confirming severe HTG-AP complicated by preeclampsia and impending preterm labor. The patient was managed through a multidisciplinary approach involving obstetrics, intensive care, endocrinology, gastroenterology, and anesthesia teams. Initial treatment included bowel rest, intravenous fluid resuscitation, insulin-dextrose infusion, and therapeutic plasma exchange (TPE). Following two sessions of TPE, serum triglyceride levels decreased markedly, accompanied by significant clinical and biochemical improvement. However, worsening abdominal pain and non-reassuring cardiotocography findings necessitated emergency cesarean delivery, resulting in the successful birth of a healthy preterm neonate. Both maternal and neonatal outcomes were favorable, and the patient was discharged in a stable condition.

This case highlights the importance of prompt recognition of HTG-AP during pregnancy, particularly when accompanied by preeclampsia, and underscores the value of multidisciplinary collaboration in optimizing maternal and fetal outcomes. Therapeutic plasma exchange may serve as an effective strategy for rapid triglyceride reduction in selected patients with severe disease.

## Introduction

Acute pancreatitis (AP) during pregnancy is a rare but potentially life-threatening condition, with an estimated incidence ranging from 1 in 1,000 to 1 in 10,000 pregnancies [1]. Although uncommon, AP is associated with significant maternal and fetal morbidity, particularly when diagnosis and treatment are delayed [1].

Diagnosis may be challenging because its clinical presentation overlaps with that of several obstetric emergencies, including preeclampsia and preterm labor, which may present with similar abdominal symptoms. Consequently, the rarity of the condition, combined with its nonspecific presentation, may delay diagnosis and increase the risk of adverse maternal and neonatal outcomes [1].

Hypertriglyceridemia-induced acute pancreatitis (HTG-AP) is the third most common cause of AP during pregnancy and is associated with severe maternal and fetal complications, including preeclampsia, preterm delivery, placental abruption, fetal loss, organ failure, and the need for intensive care support [2-3]. During pregnancy, physiological changes in lipid metabolism, including elevated estrogen levels and pregnancy-related insulin resistance, increase hepatic lipoprotein synthesis and reduce lipoprotein lipase activity, leading to progressive elevation in serum triglyceride concentrations, particularly during the third trimester. In susceptible women, severe hypertriglyceridemia may precipitate acute pancreatitis [3]. Early recognition and prompt triglyceride-lowering therapy are therefore essential to improve maternal and fetal outcomes. Therapeutic plasma exchange (TPE) has emerged as an effective treatment strategy for rapidly reducing serum triglyceride levels in severe or refractory HTG-AP, although evidence supporting its use during pregnancy remains limited [3-5].

We report the case of a 30-year-old woman at 33 weeks' gestation who developed severe HTG-AP complicated by preeclampsia. She was successfully managed using a multidisciplinary approach, including intensive care support, two sessions of TPE, and emergency cesarean delivery for non-reassuring fetal status, resulting in favorable maternal and neonatal outcomes. Although therapeutic plasma exchange has been successfully used in the management of HTG-AP during pregnancy, the coexistence of severe HTG-AP and preeclampsia presents considerable diagnostic and therapeutic challenges. This case highlights the importance of early recognition, multidisciplinary management, and timely therapeutic plasma exchange in achieving favorable maternal and neonatal outcomes.

## Case presentation

A 30-year-old woman, gravida 2 para 1, with one previous cesarean section, presented at 33 weeks' gestation. She had a medical history of type 2 diabetes mellitus, treated with metformin 1,000 mg twice daily and subcutaneous insulin, and chronic hypertension, managed with methyldopa 250 mg twice daily. She presented to the emergency department with severe headache, right upper quadrant abdominal pain associated with nausea but no vomiting, and elevated blood pressure. On examination, she was alert and oriented, afebrile, and mildly distressed because of abdominal pain, with a gravid abdomen. Urinalysis demonstrated 3+ proteinuria, and she was initially admitted with a diagnosis of preeclampsia, for blood pressure control, maternal and fetal monitoring, and further evaluation.

Initial laboratory investigations demonstrated grossly lipemic blood samples. Because of the markedly lipemic appearance, a lipid profile was obtained and revealed severe hypertriglyceridemia (>50 mmol/L) (Table 1). Serum amylase and lipase levels were elevated at 169 U/L and 328 U/L, respectively.

**Table 1:** Initial laboratory investigations at presentation Several laboratory parameters could not be analyzed because of marked lipemia of the blood samples. APTT, activated partial thromboplastin time; INR, international normalized ratio; AST, aspartate aminotransferase; ALT, alanine aminotransferase; ALP, alkaline phosphatase; LDH, lactate dehydrogenase; CRP, C-reactive protein; HbA1c, hemoglobin A1c.

Laboratory test	Result on admission	Reference range
Hemoglobin (g/dL)	10.4	12.0–15.0
White blood cell count (×10⁹/L)	7.3	4.0–10.0
Platelet count (×10⁹/L)	219	150–410
APTT (seconds)	Lipemic sample	24–31
Prothrombin time (seconds)	Lipemic sample	9.7–11.8
INR	Lipemic sample	0.8–1.2
Glucose (mmol/L)	5.0	3.3–5.5
Sodium (mmol/L)	122	133–146
Potassium (mmol/L)	3.3	3.5–5.3
Bicarbonate (mmol/L)	14	19–29
Creatinine (µmol/L)	Lipemic sample	44–80
Urea (mmol/L)	2.0	2.5–7.8
Calcium (mmol/L)	2.13	2.09–2.46
Bilirubin (µmol/L)	3	<21
AST (U/L)	5	<32
ALT (U/L)	6	<33
ALP (U/L)	82	35–104
LDH (U/L)	132	135–214
Albumin (g/L)	21	35–50
Amylase (U/L)	169	13–53
Lipase (U/L)	328	13–60
Triglycerides (mmol/L)	>50	<1.7
Total cholesterol (mmol/L)	34.2	<5.2
Beta-hydroxybutyrate (mmol/L)	4.0	0.03–0.3
Lactate (mmol/L)	1.4	0.5–2.2
CRP (mg/L)	6.8	<3.0
Urine protein (dipstick)	3+	Negative
HbA1c (%)	6.1	4.0–5.6

**Figure 1:**
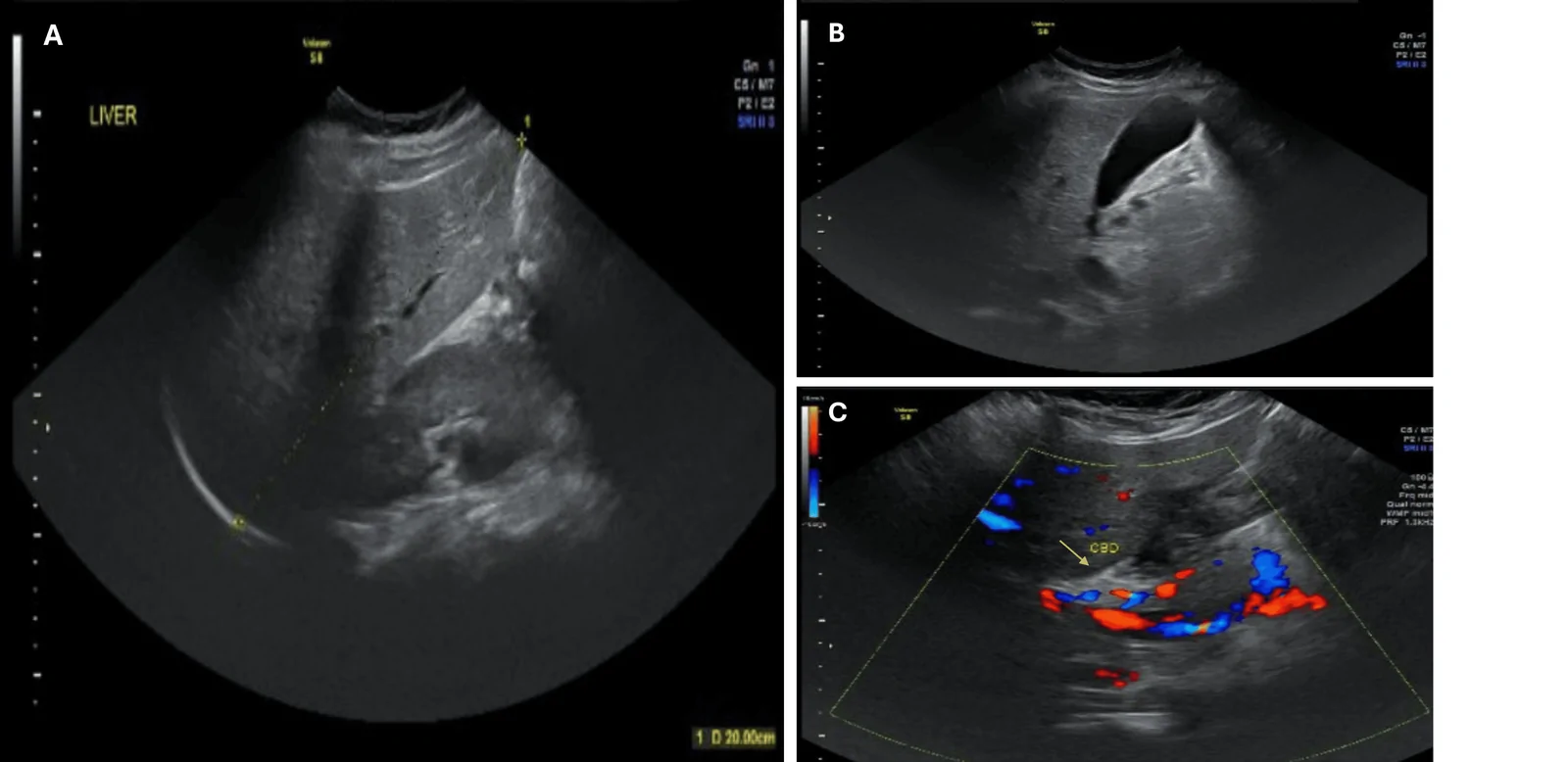
Abdominal ultrasonography demonstrating mild hepatomegaly without biliary dilatation or gallstones (A) Mild hepatomegaly (liver span approximately 20 cm) with preserved hepatic echotexture. (B) Normal gallbladder without gallstones or features of acute cholecystitis. (C) Color Doppler image demonstrating a nondilated common bile duct (arrow) without biliary obstruction.

Given her clinical presentation, the differential diagnosis included severe preeclampsia with hepatic involvement and acute pancreatitis, including both biliary and hypertriglyceridemia-induced etiologies. Abdominal ultrasonography demonstrated mild hepatomegaly without intrahepatic biliary dilatation or gallstones, making biliary pancreatitis unlikely (Figure 1). The diagnosis of acute pancreatitis was established according to the Revised Atlanta Classification based on the presence of characteristic abdominal pain and a serum lipase level greater than three times the upper limit of normal. The markedly elevated triglyceride concentration, together with the absence of gallstones or biliary obstruction, established hypertriglyceridemia as the underlying etiology.

**Figure 2:**
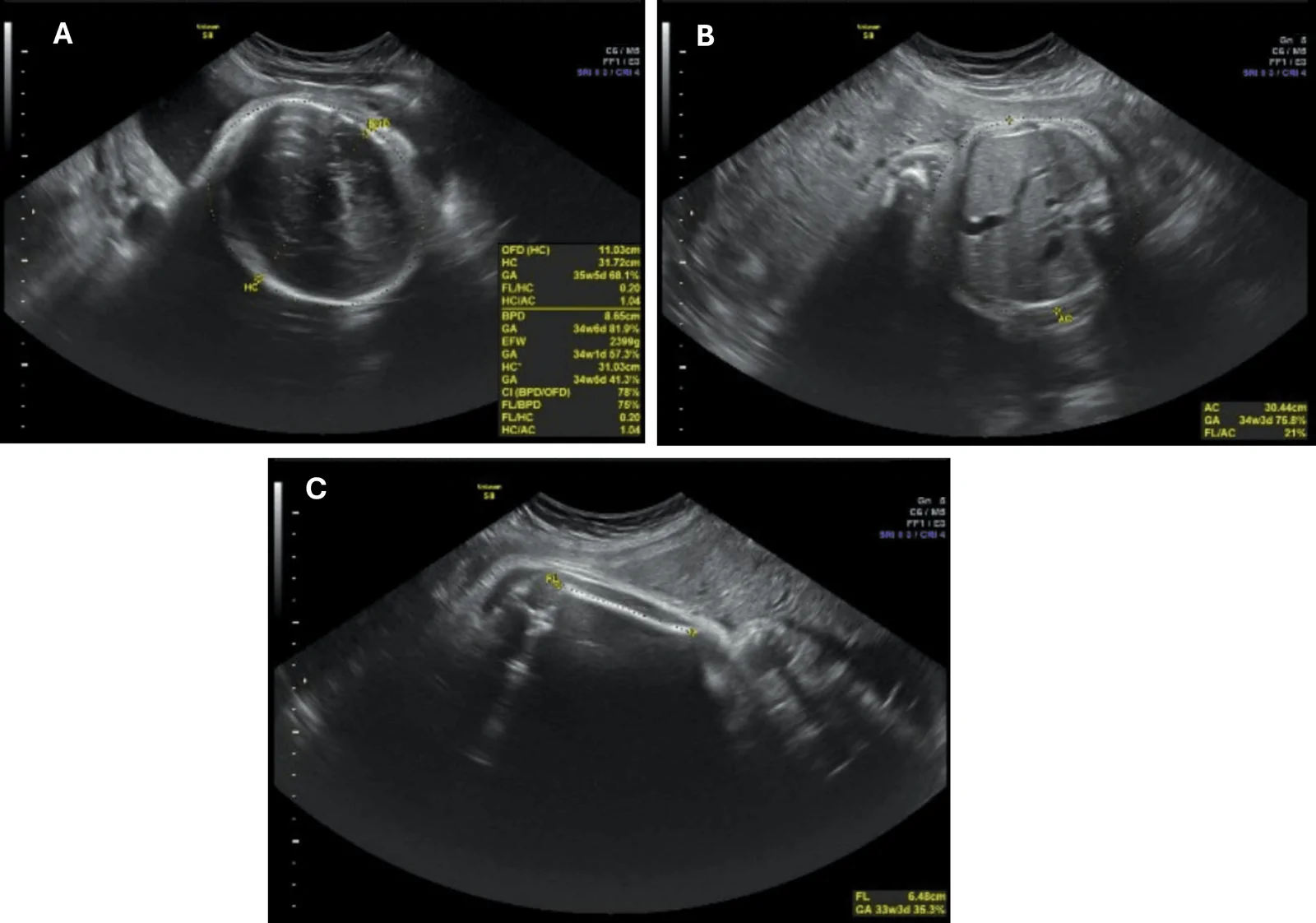
Obstetric ultrasonography demonstrating normal fetal growth parameters at 33 weeks' gestation (A) Measurement of the fetal biparietal diameter (BPD) and head circumference (HC). (B) Measurement of the fetal abdominal circumference (AC). (C) Measurement of the femur length (FL). All fetal biometric parameters were appropriate for gestational age, with no sonographic evidence of fetal growth restriction.

Obstetric ultrasonography demonstrated a single live fetus with appropriate fetal growth for gestational age, normal amniotic fluid volume, and no structural fetal abnormalities (Figure 2).

The patient's management involved a multidisciplinary approach, including the obstetrics, endocrinology, gastroenterology, intensive care, and anesthesia teams. Initial treatment consisted of intravenous fluid resuscitation, intravenous insulin/dextrose infusion, bowel rest, and urgent therapeutic plasma exchange to achieve rapid triglyceride reduction. In case of clinical deterioration, pregnancy termination was considered as part of the management strategy.

On day 2 of admission, the patient was transferred to the intensive care unit (ICU), where the first TPE session was performed. Following the first session, her serum triglyceride level decreased to 36.6 mmol/L, her abdominal pain improved, and serial fetal heart rate monitoring and cardiotocography (CTG) remained reassuring.

**Figure 3:**
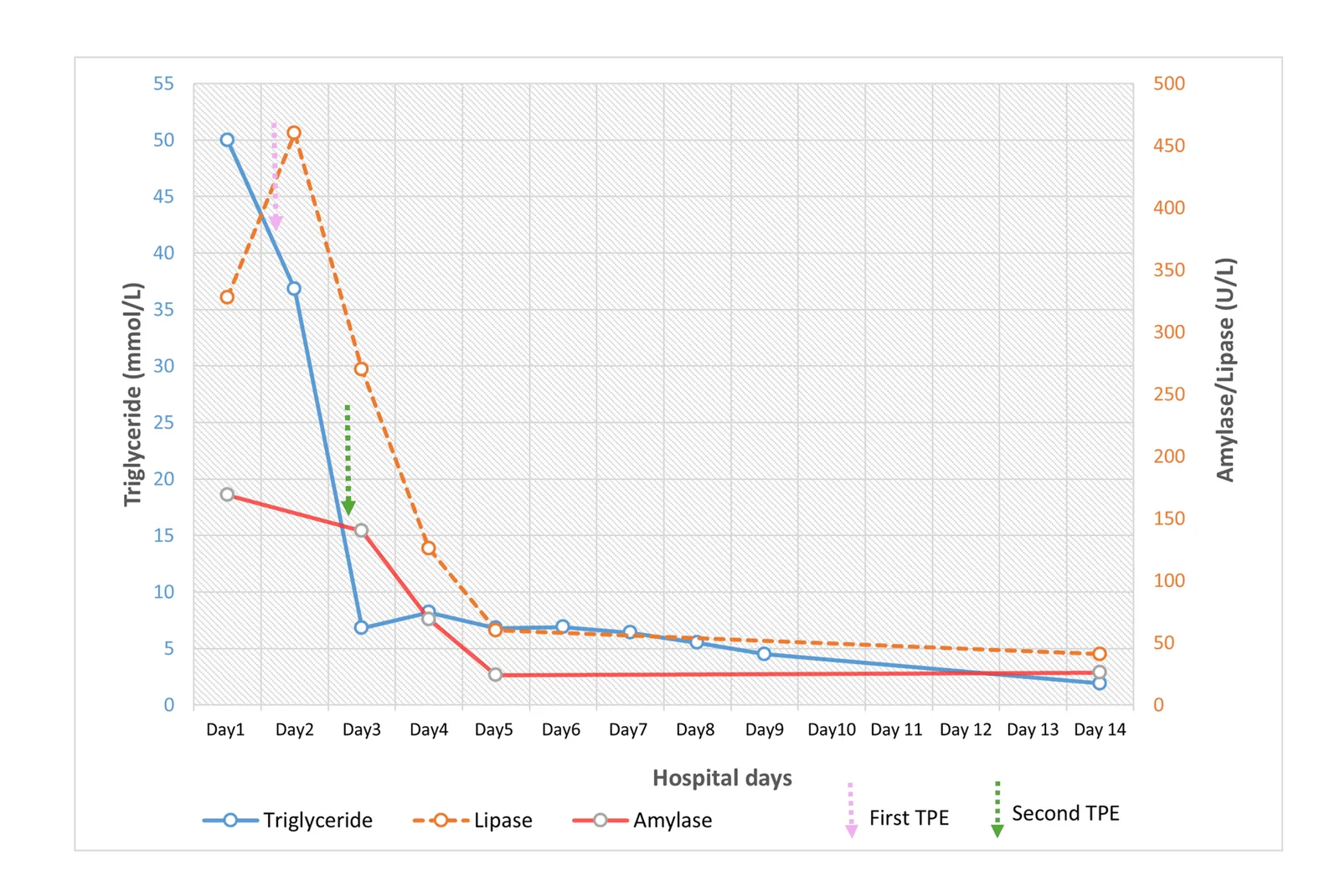
Serial changes in serum triglyceride, lipase, and amylase levels during hospitalization following therapeutic plasma exchange Serum triglyceride levels decreased markedly after two TPE sessions, with concurrent improvement in pancreatic enzyme levels. Arrows indicate the timing of each TPE session. TPE, therapeutic plasma exchange

A second session of TPE was conducted on day 3, after which triglyceride levels dropped to 6.8 mmol/L. The trend of serum triglyceride and pancreatic enzyme levels following TPE is shown in Figure 3. As her clinical condition improved, she was allowed to have sips of water, and omega-3 fatty acids (2 g twice daily) were initiated.

During the early morning of day 4, the patient developed worsening abdominal pain and non-reassuring CTG findings, including early and variable decelerations. An emergency cesarean section was therefore performed under spinal anesthesia. A healthy male infant weighing 2.74 kg was delivered, with Apgar scores of 8 and 9 at one and five minutes, respectively. The neonate was admitted to the neonatal intensive care unit (NICU) for observation because of prematurity. No significant neonatal complications occurred, and the infant was discharged in good clinical condition. During the patient's cesarean section, thick yellowish intraperitoneal fluid was observed, consistent with pancreatitis-related inflammatory exudate.

Postoperatively, the patient remained clinically stable. On day 5, her serum triglyceride level dropped to 6.9 mmol/L, and treatment with intravenous insulin/dextrose infusion and omega-3 fatty acids was continued. By day 6, the serum triglyceride level had further decreased to 5.5 mmol/L, the intravenous insulin/dextrose infusion was stopped, and the patient was transferred to the high-dependency unit (HDU). A subcutaneous sliding-scale insulin regimen was initiated for glycemic control, and omega-3 supplementation was maintained.

The patient remained clinically stable. On day 9, her serum triglyceride level had decreased to 4.5 mmol/L, and she was discharged home on day 11 in stable condition.

One week after discharge, she was readmitted with recurrent abdominal pain. Contrast-enhanced computed tomography demonstrated a 15 × 5 × 15 cm peripancreatic fluid collection without evidence of pancreatic necrosis. She was managed conservatively with intravenous antibiotics and supportive care, resulting in gradual clinical improvement without the need for percutaneous or surgical drainage. She was discharged four days later in good condition, with a scheduled follow-up in the endocrinology and gastroenterology clinics.

## Discussion

Acute pancreatitis is an uncommon but serious condition during pregnancy, with an estimated incidence ranging from 1 in 1,000 to 1 in 10,000 pregnancies, and is associated with adverse maternal and fetal outcomes. Hypertriglyceridemia accounts for approximately 4% of cases and is the third most common cause of AP during pregnancy, after gallstones and alcohol [1,2]. During pregnancy, high estrogen levels increase lipoprotein synthesis, and increased insulin resistance decreases the activity of lipoprotein lipase. Consequently, triglyceride levels can increase to two to four times above normal levels in late gestation, particularly in concurrent gestational diabetes [3].

Severe gestational hypertriglyceridemia usually refers to a plasma triglyceride level greater than 11.4 mmol/L, and it is associated with an increased risk of developing acute pancreatitis. Elevated triglyceride levels during pregnancy have also been linked with preeclampsia, preterm birth, gestational diabetes [6,7], and neonatal complications such as prematurity, low birth weight, and stillbirth [3]. Early diagnosis and rapid treatment are crucial and associated with better maternal and fetal outcomes.

The pathogenesis of hypertriglyceridemia-induced acute pancreatitis remains incompletely understood. It is proposed that hydrolysis of triglycerides accumulating around the pancreas by pancreatic lipase results in the accumulation of high levels of free fatty acids, which have cytotoxic effects on acinar cells and the capillary endothelium. Another mechanism suggests that elevated levels of chylomicrons can lead to capillary obstruction, ischemia, and acidosis. Within this acidic environment, free fatty acids can activate trypsinogen and initiate acute pancreatitis [8,9].

According to the Revised Atlanta Classification, our patient was classified as having moderately severe acute pancreatitis, as she developed a local complication (pancreatic fluid collection) in the absence of persistent organ failure [10]. Severe hypertriglyceridemia may interfere with laboratory measurements because marked lipemia can produce analytical errors, including pseudohyponatremia and difficulty interpreting several biochemical parameters. In our patient, the grossly lipemic blood samples prompted lipid profile testing, which facilitated the diagnosis of HTG-AP [11].

Our patient initially had a serum sodium concentration of 122 mmol/L. In the setting of severe hypertriglyceridemia, this finding likely represented pseudohyponatremia resulting from lipemic interference with indirect ion-specific electrode measurement rather than true sodium depletion. Recognition of this laboratory artifact is important to avoid inappropriate clinical management [12].

Management of HTG-AP is challenging and requires supportive care, including aggressive intravenous hydration, bowel rest, and adequate analgesia, together with rapid reduction of serum triglyceride levels [13]. In pregnancy, therapeutic options for triglyceride lowering are limited. Among oral lipid-lowering agents, omega-3 fatty acids are considered a safe option during pregnancy [14]. The use of fibrates is limited because few data are available from well-designed studies; statins are generally avoided because of their potential teratogenic effects, although existing data remain controversial [1]. Intravenous insulin infusion may also be used to increase lipoprotein lipase activity, thereby enhancing triglyceride clearance [1].

In cases of markedly elevated triglyceride levels or multiorgan dysfunction, TPE may be considered [4] because it rapidly lowers serum triglyceride levels by removing triglycerides and chylomicrons and may provide additional benefit through the removal of inflammatory mediators and cytokines [3,4]. Several case reports and small studies have demonstrated the efficacy and safety of TPE during pregnancy [4,15]. Although intravenous insulin infusion is an effective medical therapy for lowering triglyceride levels, TPE was selected in our patient because of the markedly elevated triglyceride level (>50 mmol/L), the presence of acute pancreatitis, and pregnancy, where rapid triglyceride reduction was considered essential to minimize maternal and fetal complications.

In our patient, serum triglyceride levels decreased from >50 mmol/L to 36.8 mmol/L after the first TPE session (approximately 26% reduction) and subsequently to 6.8 mmol/L after the second session, representing an overall reduction of at least 86%. These findings are consistent with previous reports demonstrating that TPE can reduce triglyceride concentrations by 50%-80%, particularly in patients with severe or refractory hypertriglyceridemia [3,16,17]. Notably, betamethasone was not administered for fetal lung maturation because of concerns that corticosteroids may exacerbate hypertriglyceridemia by increasing triglyceride synthesis. In addition, pregnancy termination may be considered in cases of progressive maternal or fetal deterioration, when continuation of pregnancy poses a significant risk.

The coexistence of severe hypertriglyceridemia-induced acute pancreatitis and preeclampsia represents one of the most important aspects of this case. Gestational hypertriglyceridemia has been associated with an increased risk of preeclampsia [18]. The mechanisms underlying this association are not fully understood but are thought to involve oxidative stress-mediated endothelial dysfunction, insulin resistance with reduced lipoprotein lipase activity leading to impaired triglyceride clearance, and free fatty acid-induced endothelial injury [18]. While maternal and fetal mortality rates for AP in pregnancy are historically high - reported up to 7% and 60%, respectively - advances in early diagnosis and management have significantly improved outcomes. The recent literature reports maternal mortality rates as low as 0% and fetal mortality as low as 3% [18]. Despite these improvements, acute pancreatitis in pregnancy is still associated with high risks of maternal and fetal adverse outcomes such as preterm delivery, fetal demise, low birth weight, and prematurity [19].

Our case demonstrates that therapeutic plasma exchange was associated with rapid triglyceride reduction and favorable maternal and neonatal outcomes. Although evidence remains limited to observational studies and case reports, TPE may be considered in selected patients with severe HTG-AP who require rapid triglyceride reduction, particularly in the presence of clinical deterioration or organ dysfunction. This case also highlights the importance of early multidisciplinary collaboration, involving specialists in obstetrics, intensive care, gastroenterology, endocrinology, and anesthesiology, in achieving successful maternal recovery and favorable neonatal outcomes.

## Conclusions

Hypertriglyceridemia-induced acute pancreatitis is an uncommon but serious condition during pregnancy. Rapid recognition and treatment with a multidisciplinary team approach are crucial for improving maternal and neonatal outcomes. Effective management requires supportive therapy alongside rapid reduction of serum triglyceride levels. Therapeutic plasma exchange may be considered in selected patients with severe HTG-AP requiring rapid triglyceride reduction, particularly in the presence of clinical deterioration or organ dysfunction.
